# Cell‐based crop phenotyping for future climates

**DOI:** 10.1111/nph.71397

**Published:** 2026-07-09

**Authors:** Sergey Shabala, Ping Yun, Zhong‐Hua Chen, Meixue Zhou, Min Yu, Nadia Bazihizina

**Affiliations:** ^1^ International Research Centre for Environmental Membrane Biology Foshan University Foshan 528041 China; ^2^ School of Biological Sciences and ARC Training Centre for Smart and Sustainable Horticulture University of Western Australia Crawley WA 6009 Australia; ^3^ School of School of Agriculture, Food and Wine, Waite Research Institute Adelaide University Glen Osmond SA 5064 Australia; ^4^ Tasmanian Institute of Agriculture University of Tasmania Launceston TAS 7250 Australia; ^5^ Department of Biology University of Florence 50121 Florence Italy

**Keywords:** abiotic stress, climate change, drought, salinity, soil flooding, spatial omics

## Abstract

Abiotic stress tolerance has been significantly weakened in modern crops during the domestication process. Regaining tolerance has become a critical task in light of current climate trends and their impact on global food security. Abiotic stress tolerance is an extremely complex trait and is conferred at various levels of plant functional organization and developmental stages, with regulatory mechanisms operating across multiple scales, from individual cells to tissues and the entire plant. The emergence of advanced molecular tools such as single‐cell RNA sequencing and spatial omics technologies has revolutionized the field, advancing our understanding of plant responses to hostile environments. However, the implementation of this knowledge in crop breeding programmes is handicapped by the lack of appropriate phenotyping platforms. Here, we argue that current phenotyping methods may be excellent tools for functional validation of previously discovered traits but have limited predictive value in stress biology. We also propose that bridging the mismatch between omics technologies and phenotyping is the only way to account for cell‐specific operation of key genes conferring stress tolerance and implementing them in breeding programmes. Some practical examples using cell‐based phenotyping tools such as fluorescence dyes or electrophysiological methods are given, and current limitations and prospects of cell‐based phenotyping are discussed.

## Graphical abstract

Flow‐chart diagram illustrating the proposed pathway for improving the throughput of fluorescence imaging‐based phenotyping by using robotics and machine learning (ML) approaches.

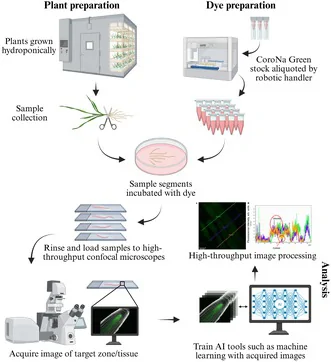

## Climate trends and global good security

Climate change comes with a massive cost to agriculture. The three years spanning 2023 to 2025 were the warmest consecutive years on record (Tollefson, 2025; WMO, 2026), and this warming trend is unlikely to change soon. While elevated temperatures *per se* exert substantial negative impacts on agricultural productivity (White *et al*., 2023), climate change also increases the frequency and intensity of many extreme hydrological events (Raghuraman *et al*., 2024; Toreti *et al*., 2024; Liu *et al*., 2025; UNCCD, 2025). This comes with massive financial losses to the industry. In Southern Africa, the El Niño phenomenon in 2023 induced droughts that led to agricultural production losses of up to 70% in some areas (FAO, 2025). In Brazil, prolonged droughts in 2020–2023 led to estimated losses of US$ 8.2 billion in soybean production, alongside severe reductions in yields of other cash crops (Nóia‐Júnior *et al*., 2025). By contrast, the 2023–2025 period also witnessed devastating floods across multiple regions world‐wide. In many cases, these floods were amplified by preceding droughts, creating long‐term challenges for agricultural productivity (Council of Europe, 2024; Cremonini *et al*., 2024).

Adding another layer of complexity is the issue of soil salinization. With the increasing number of hot days, agricultural food production relies heavily on irrigation (Liu *et al*., 2020; Palmgren & Shabala, 2024), with agricultural water consumption increasing almost sixfold in the last 100 years (https://ourworldindata.org/water‐use‐stress). This is not surprising, as in many parts of the world irrigation‐based productive systems are an order of magnitude more effective than rainfed systems (Palmgren & Shabala, 2024). Given that the arid and semi‐arid regions already account for over 40% of agricultural land (IWG Drought, 2020), the reliance on irrigation is only going to increase. However, this comes with a caveat of adding between 3 and 6 tons of salt per hectare of irrigated land every year (Liu *et al*., 2020). This leads to salinity build‐up in the rhizosphere, ultimately making land unsuitable for producing most major crops. A recent meta‐analysis demonstrated that, given the current trends of land salinization, wheat yield could decline by up to 70% by 2050, while rice production will be simply unfeasible unless new salt‐tolerant varieties are developed (Sun *et al*., 2025). Given that these two cereal crops collectively provide over 40% of total human calorie intake, the implications for global food security are profound. The economic costs are also substantial, with estimated overall losses in crop production from climate‐driven abiotic stresses exceeding US$170 billion per year (Razzaq *et al*., 2021). Taken together, improving abiotic stress tolerance in crops is an urgent task.

## Complexity of abiotic stress tolerance and a need to account for cell specificity

Abiotic stress tolerance is an extremely complex trait, both physiologically and genetically, and is conferred at various levels of plant functional organization. Indeed, regulatory mechanisms operate across multiple scales, from individual cells to tissues and entire organs, collectively shaping development and adaptive responses to fluctuating environmental conditions (Dinneny, 2015; Feng *et al*., 2016). As a result, plant responses to abiotic stress involve highly specific gene expression changes tailored to individual cell types, often regulated by epigenetic modifications like histone methylation and acetylation.

To start with, adaptation to a hostile environment requires a tight coordination of operation of aboveground and belowground organs; this implies an efficient root‐to‐shoot communication that is achieved by various physical (electric and hydraulic signals, propagating Ca^2+^ and reactive oxygen species (ROS) waves), chemical (assimilates, hormones, peptides and nutrients), and molecular (proteins and RNA) signals (Shabala *et al*., 2016; Singh *et al*., 2024; Gul *et al*., 2026). Within each of these organs, different cell types possess different abilities to sense and decode these signals and, therefore, play different roles while dealing with stress, up‐ and/or downregulating a specific set of genes to account for this need. Taking roots as an example: these represent a mixture of at least 27 functionally diverse cell types, each perceiving and responding to environmental stresses differently (Dinneny *et al*., 2008; Kawa & Brady, 2022). For example, in rice roots abscisic acid (ABA) biosynthesis genes were upregulated by soil compaction predominantly in the inner cell layers, whereas ABA responders were activated in outer cell layers (Zhu *et al*., 2025); genes from the expansin family were also specifically upregulated in the exodermis and cortex, while increased expression of genes involved in N and P metabolism was observed in outer cell layers. The top 10 genes induced by salt stress in wheat were related to osmotic and ion homeostasis and significantly enriched in the endodermis, root border cells and root hairs (Du *et al*., 2025); conversely, epidermis, cortex, xylem parenchyma, phloem and root cap cells showed enrichment for oxidative stress mitigation. Transcriptional profiles of a key gene involved in ROS scavenging *GSTU25* encoding glutathione S‐transferase were much higher in the root cap of nonextremophyte species, while in extremophytes, its expression becomes enriched in the endodermal cells (Wang *et al*., 2024), explaining differential abiotic stress tolerance between these two groups of species. Also, root hairs exhibited the highest number of differentially expressed genes (DEGs) among all cell types while exposed to salt stress (Du *et al*., 2025).

The cell‐ and tissue‐specificity of stress responses goes beyond transcriptional regulation and is also manifested at the functional level. Salt stress‐induced changes in root metabolic profiles were much greater in the root apex than in the mature root zone (Shabala *et al*., 2016), and changes in root proteome in drought‐affected plants were more pronounced in the apex than in the proximal zone (Wang *et al*., 2021). In Arabidopsis, treatment with aluminium induced an increase in auxin content in the root apical meristem zone and transition zone, whereas in the elongation zone the auxin content was decreased beyond the level required for adequate growth, due to the Al‐inhibited PIN‐FORMED 2 endocytosis specific to this zone (Tao *et al*., 2023). Wang *et al*. (2021) identified a postmeristematic K^+^‐sensing niche (KSN) that allows plants to sense K^+^ deprivation. By integrating K^+^ and Ca^2+^ signalling, cells in this KSN activate the CIF‐RLK/RLCK‐NOX (Casparian Strip Integrity Factor – Receptor‐Like Kinase/Receptor‐Like Cytoplasmic Kinase – NADPH oxidase) module; together with auxin‐mediated modulation of meristem activity and regulation of root hair growth (Dindas *et al*., 2018), this mediates plant adaptation to K‐impoverished soils. Thus, this small group of cells functions as a hub facilitating multidirectional signalling to coordinate spatially separated, diverse downstream responses, including regulation of stem‐cell activity, modulation of endodermis differentiation and adjustment of gene transcription and ion transport.

## Accounting for cell‐specificity by modern omics

In the last decade, the emergence of single‐cell RNA sequencing (scRNA‐seq) and, more recently, spatial omics technologies, has revolutionized the field of cellular biology. In mammalian research, these approaches have enabled the investigation of gene expression, epigenomic regulation and cellular heterogeneity across development, ageing and disease at an unprecedented resolution (Vandereyken *et al*., 2023; Liu *et al*., 2024). This is also reflected in the exponential growth of publications adopting these approaches, with > 31 900 studies published since 2020 in Scopus (see Supporting Information Notes S1). A notable example of their impact is cancer biology, where single‐cell and spatial profiling have revealed complex cell–cell communication networks between tumour cells and the surrounding microenvironment. These insights have directly informed the development of targeted therapeutic drugs, including drugs targeting specific signalling checkpoints (Dominguez *et al*., 2020; Upasana *et al*. 2022; Lee & Lee, 2025; Liu *et al*., 2026), and biomarkers for early cancer detection (e.g. combined detection of GAS2L1 and EPCAM1; Zhu *et al*., 2020) currently tested in multiple clinical trials.

In plant research, the adoption of single‐cell and spatial omics has progressed at a considerably slower pace than in mammalian research, with 486 and 156 studies respectively published since 2020 in Scopus (see Notes S1 for search criteria). Nevertheless, this is a rapidly changing field, as highlighted by several recent reviews (Yu *et al*., 2023; Zhu *et al*., 2023; Grones *et al*., 2024; Barmukh *et al*., 2025; Ferreras‐Garrucho *et al*., 2025; Nobori, 2025; Yao *et al*., 2025, and references therein). To date, single‐cell and spatial transcriptomics have been used predominantly to understand various aspects of plant development and their interactions with pathogens and microorganisms (Barmukh *et al*., 2025; Nobori, 2025). The studies related to abiotic stress responses are not as numerous but are rapidly gaining momentum (Tao *et al*., 2026 and references therein) and have facilitated the identification of tissue‐specific genes and pathways currently mediating plant adaptation to hostile environments.

There is, however, currently very little quantitative information on the success rates of using these advanced omics approaches for cultivar development and/or integration into commercial breeding programmes. Although this gap may in part be due to their relatively recent emergence, scaling up these laboratory breakthroughs into practical agricultural solutions remains a major challenge, often referred to as the ‘Agri‐Tech Valley of Death’ (cf. Barmukh *et al*., 2025; Pacheco‐Ruiz *et al*., 2025). Beyond their high implementation costs, which constrain their widespread adoption, the major constraint in this process can be attributed to the lack of suitable phenotyping platforms that can account for cell‐ and tissue‐specific operation of key genes, proteins and metabolites mediating plant adaptive responses to hostile environments.

## Phenotyping as a bottleneck in breeding programmes

Historically, the three major pillars of plant phenotyping are: its cost; convenience and simplicity of use; and its high‐throughput capacity. All three factors are closely interrelated. As plant breeders are interested in methods that will allow the screening of thousands of genotypes per day, the methods used should be fast, require minimal training and come at relatively low cost. The latter is especially true for developing countries. As a result, many breeders still rely on visual scoring (e.g. various damage indices) as a preferred method.

Over the past two decades, this traditional phenotyping has been gradually replaced by more sophisticated robotic‐based imaging platforms. One of the early examples is PHENOPSIS, an automated system for monitoring Arabidopsis phenotypes in response to drought, demonstrating that platform engineering could convert classical assays to standardized pipelines (Granier *et al*., 2006). As genomics accelerated in the late 2000s, phenotyping was recognized as a bottleneck, and various tools such as imaging, thermal infrared, hyperspectral sensors, automated weighing/irrigation and high‐throughput data handling have emerged for multiscale (organ to canopy) and multimodal (structure and physiology) phenotyping (Furbank & Tester, 2011; Fiorani & Schurr, 2013).

The second major shift was bringing phenomics to field conditions. Field high‐throughput phenotyping with spectral and imaging approaches enabled rapid plot‐level assessment (Araus & Cairns, 2014). Practical implementations, such as remote sensing buggies or fixed robotic systems, were used for plot phenotyping in a tractable manner or for monitoring experimental fields (Deery *et al*., 2014; Virlet *et al*., 2016). Over time, the obstacles were not only about sensing but also about converting these data into workable biological knowledge and integrating the data with models (Tardieu *et al*., 2017). This evolution has been accompanied by the growth of research infrastructures and commercial suppliers of plant phenotyping (Table 1; Araus & Cairns, 2014; Yang *et al*., 2020; Awada *et al*., 2024). Community networks and distributed infrastructures aim to lower barriers by sharing standards, providing access to facilities and providing training. The International Plant Phenotyping Network (IPPN), founded in 2015, explicitly connects major phenotyping centres world‐wide and stakeholders across academia and industry. Some of the leading facilities are listed in Table 1. On the commercial side, companies such as LemnaTec, Phenospex, Photon Systems Instruments/PlantScreenTM and PlantDitech exemplify the integration of hardware and software approaches with computerized phenotyping. The benefits are evident, from a financial perspective, when such systems/facilities are used for phenotyping large populations or for shortened selection cycles in breeding. In addition, public investment also contributes to the development of phenotyping infrastructure at the national scale; for example, the Australia Plant Phenomics Network (APPN) recently received AU$60M core funding from the Australian Government National Collaborative Research Infrastructure Strategy (NCRIS) to expand controlled‐environment and field phenotyping capacity.

**Table 1:** Leading plant phenomics facility/programme/company.

City (country)	Plant phenomics facility/programme/company
Aachen (DEU)	LemnaTec
Adelaide (AUS)	The Australian Plant Phenomics Network
Athens (USA)	Institute for Integrative Precision Agriculture, University of Georgia
Auzeville‐Tolosane (FRA)	The PhenoToul, INRAE
Barcelona (ESP)	Integrative Crop Ecophysiology Group, University of Barcelona
Beijing (CHN)	PhenoTrait
Bonn (DEU)	PhenoRob
College Station (USA)	The Texas A&M Plant Growth and Phenotyping Facility
Davis (USA)	Digital Agriculture Laboratory, University of California Davis
Drásov (CZE)	PlantScreen™, Photon Systems Instruments
East Lansing (USA)	Center for Advanced Algal and Plant Phenotyping, MI State University
Eeklo (BEL)	Weighing, Imaging & Watering Machines for Plant Phenotyping
Heerlen (NLD)	Phenospex
Ithaca (USA)	BTI Plant Phenotyping Facility, Cornell University
Julich (DEU)	Jülich Plant Phenotyping Center, Jülich Forschungszentrum
Lincoln (USA)	Plant Phenotyping Facility, University of Nebraska‐Lincoln
Los Banos (PHL)	International Rice Research Institute Bio‐Innovation Center (IRRI)
Madison (USA)	Digital Agriculture, Biological Systems Engineering, University of Wisconsin
Montpellier (FRA)	Montpellier Plant Phenotyping Platforms
Nanjing (CHN)	Plant Phenomics Research Center, Nanjing Agricultural University
Nottingham (GBR)	PhenomUK Research Infrastructure
Potsdam (DEU)	Phenotypic plasticity in plants, Collaborative Research Centre 1644
Pullman (USA)	WSU Plant phenomics, Washington State University
Rome (ITA)	Phenomics Platform, CIAT
Saskatoon (CAN)	Plant Phenotyping and Imaging Research Centre, University of Saskatchewan
Seeland (DEU)	Phenotyping Infrastructure, IPK Leibniz Institute of Plant Genetics and Crop Plant Research
St. Louis (USA)	Phenotyping Facility, Donald Danforth Plant Science Center
St. Paul (USA)	Precision Agriculture Center, University of Minnesota
Tokyo (JPN)	Laboratory of Field Phenomics, University of Tokyo
Urbana‐Champaign (USA)	RIPE High‐Throughput Phenotyping Facility, University of Illinois Urbana‐Champaign
Versailles (FRA)	Phenome‐Emphasis, INRAE
Vienna (AUT)	Vienna BioCenter Core Facilities
Wageningen (NLD)	The Netherlands Plant Eco‐phenotyping Centre , Wageningen University & Research
West Lafayette (USA)	Phenotyping facility, Purdue University
Wuhan (CHN)	Crop Phenotyping Center, Huazhong Agricultural University
Yavne (ISR)	Plant Phenotyping Platform, PlantDitech
Zurich (CHE)	Crop Science, ETH Zurich

Country names are defined by three‐letter country codes according to the ISO 3166 standard. Modified from Awada *et al*. (2024).

Currently, various types of techniques are employed in plant phenotyping, such as photogrammetry (An *et al*., 2017), microscopy (He *et al*., 2025), 3D laser scanning (Ziamtsov & Navlakha, 2019), spectral imaging (Liu *et al*., 2020), remote sensing light detection and ranging (LiDAR) (Forero *et al*., 2022), drones (Gano *et al*., 2024) and AI‐assisted tools (e.g. machine learning; Zhou *et al*., 2021). Such combinations, without any doubt, have made the phenotyping process easier and faster, with higher throughput and more automation. Although several issues remain (e.g. limits on the data that can be obtained; the accuracy of computer models), they are mostly technical barriers that are getting resolved (Gibbs *et al*., 2018; Liu *et al*., 2019; Ziamtsov & Navlakha, 2019). A more serious question is whether whole‐plant‐based phenotyping can capture the tissue‐ and cell‐specific operation of key genes conferring abiotic stress tolerance?

## Can whole‐plant phenotyping account for cell‐specific gene operation?

The main driving force behind developing the above image‐based automated platforms was a need to enable the high throughput of screening to meet breeders' demands. However, at that time, breeding for stress tolerance was not high on the agenda, with various yield and quality‐related traits given priority. From this point of view, such image‐based technology platforms represented an ideal tool for rapidly assessing plant phenology, yield stability, developmental trade‐offs or genotype × environment interactions. However, as global agriculture is approaching a tipping point due to climate‐driven constraints on food production, breeding for improved abiotic stress tolerance has moved high on the agenda. It is widely recognized that abiotic stress tolerance is significantly weakened during domestication (Palmgren *et al*., 2015; Gilliham *et al*., 2017; Menguer *et al*., 2017). For example, a high affinity potassium transporter TmHKT1;5‐A, that encodes a high‐affinity Na^+^ transporter at the plasma membrane, contributes to salt tolerance in Einkorn (*Triticum monococcum*), a diploid ancestor of both tetraploid durum wheat (*T. turgidum* ssp. *durum*) and hexaploid bread wheat (*T. aestivum*) but is no longer present in durum and bread wheat (Munns *et al*., 2012). Similarly, domestication of tomato (*Solanum lycopersicum*) resulted in a loss of functionality of a potassium transporter SlHAK20 and reduced salinity tolerance that was present in its ancestral relative species *S. pimpinellifolium* (Wang *et al*., 2020). Such a shift in breeding focus required phenotyping methods adopted for this purpose. However, the success of this adoption was rather limited. For example, the discovery of the plant *NHX1* (Apse *et al*., 1999) gene that confers Na^+^ sequestration in the vacuole was a result of sequencing the *Arabidopsis thaliana* genome and then comparing it for sequences similar to known animal and bacterial sodium/proton exchangers performing a similar role. Similarly, the aluminium‐activated malate transporter ALMT1 (Sasaki *et al*., 2004), that confers plant tolerance to soil acidity, was discovered by screening plant roots for their ability to secrete malate, thus modulating rhizosphere pH and reducing the amount of soluble Al^3+^. Another example is the discovery of genes from the *EPF* (*Epidermal Patterning Factor*) family that control stomatal density and directly affect plant water use efficiency. These genes were discovered through a series of genetic screening and protein overexpression patterns looking at stomata patterning (Caine *et al*., 2016), and whole‐plant phenotyping was then used only to validate the role of EPF homologues in drought tolerance in various species like rice (Lu *et al*., 2019) or barley (Hughes *et al*., 2017) with modified EPF expression levels. Thus, whole‐plant‐based phenotyping appears to be an ideal tool for *validating* overall plant performance under stress conditions but with a limited role *in direct discovery* of new genes conferring abiotic stress tolerance in plants. This is hardly surprising, as whole‐plant methods integrate performance of hundreds and thousands of genes potentially contributing to plant adaptive responses to environmental constraints and do not account for their cell‐specific operation. For example, a total of 1143 and 6380 DEGs were identified in pear rootstock in response to cold and drought conditions, respectively (Wang *et al*., 2025). Given these large numbers, revealing the relative contribution of these genes towards stress tolerance via whole‐plant phenotyping is an unrealistic task.

The same is true for other abiotic stresses. Exposing Arabidopsis roots to hypoxia resulted in the identification of 586 DEGs (Hill *et al*., 2026); however, only 43 were expressed in 10 or more cell types while others were unique and occurred only in specific cell types. Even more striking was the difference in responses to boron deficiency in pea plants (Chen *et al*., 2024), with 3402 DEGs reported for mesophyll cells, 2640 for leaf epidermis and 2246 for guard cells. Of these, *c*. 150 DEGs were shared by different cell types; such large number make it impossible to assess their relative contribution by phenotyping at the whole‐plant level.

Equally problematic are less advanced and more traditional phenotyping methods based on various biochemical and growth assays. Measuring seed germination rate or a percentage of germinated seeds comes at almost no cost, requires little infrastructure and is, therefore, highly attractive. The search on Web of Science returns nearly 9000 papers attempting to use seed germination and early seedling growth as a proxy for salt tolerance. However, it was also shown that seed germination ability does not necessarily correlate with overall salinity stress tolerance (Munns & James, 2003; Munns *et al*., 2006; de la Reguera *et al*., 2020), as other mechanisms that become involved at the later developmental stages, such as control of xylem salt loading, stomata operation and Na sequestration in leaf vacuoles, play much more important roles. Yet, the temptation for a shortcut is far too strong, and the stream of such papers continues. The same is true for enzymatic antioxidant (AO) assays. As many stresses lead to excessive accumulation of ROS, enzymatic AOs have often been advocated as suitable proxies for stress tolerance by numerous authors (reviewed in Xu *et al*., 2025). However, the constitutively higher AO activity may interfere with stress‐induced ROS signalling (Fichman & Mittler, 2021; Fichman *et al*., 2023) and can be a disadvantage for plant stress tolerance. Thus, stress‐induced ROS accumulation and regulation of redox balance should be considered in a very strict time‐ and cell‐specific context (Xu *et al*., 2025).

Plant phenotyping for salinity stress tolerance by analysing bulk shoot or root Na^+^ content is another example of a traditional misconception (Palmgren & Shabala, 2024), as this method fails to account for differential plants' ability for the vacuolar Na^+^ sequestration – a key trait conferring salinity tolerance in halophytes (Flowers & Colmer, 2008; Shabala, 2013). Indeed, *Atriplex* species may accumulate between 8% and 12% Na^+^ in the leaf lamina and show no symptoms of growth inhibition while grown at half‐strength seawater salinity (Panta *et al*., 2014). However, rice plants die at 25% of that level while accumulating < 2% NaCl (Ishikawa & Shabala, 2019). In line with this, as several studies with salt‐tolerant glycophytes have shown, salt tolerance does not necessarily correlate with shoot salt exclusion (Munns & James, 2003; Genc *et al*., 2007; Busoms *et al*., 2018), and there have been recent calls to reassess the relative importance traditionally attributed to salt exclusion as a key determinant of salt tolerance (Bazihizina *et al*., 2019; Garcia‐Daga *et al*., 2025).

## Novel gene discovery will be difficult without cell‐based phenotyping

It is becoming increasingly evident that the ‘silver bullet approach’ – namely an attempt to improve abiotic stress tolerance by modifying operation of just one gene – is not likely to deliver the anticipated outcome in breeding programmes. This is hardly surprising, given hundreds of reported DEGs and their tissue‐ and cell‐specific patterning. The omics data *per se* is only a starting point, but the functional roles of these genes need to be validated. While many of them may represent a general feedback response of the organism to the stress‐related disturbance, others may represent previously unexplored targets for breeding programmes. Thus, efficient phenotyping platforms must not only validate performance of modified organism harbouring altered gene(s) but also possess predictive power for quantifying the relative contribution of the gene of interest towards stress tolerance. This is a highly challenging yet doable task.

The above notion is further illustrated by several examples. Until now, most attempts to improve salinity stress tolerance in crops were made by targeting salt overly sensitive 1s (SOS1s) and HKT1s. The first operates as an Na^+^/H^+^ exchanger (Zhao *et al*., 2020) and is involved in the removal of Na^+^ from the cytosol, whereas the HKT1 transporter is located in the xylem parenchyma and operates in Na^+^ retrieval from the shoot (Byrt *et al*., 2007; Kotula *et al*., 2020). However, overexpressing SOS1 genes comes with a caveat of increased loading of Na^+^ into the xylem and its delivery to the shoot, as well as numerous pleiotropic effects; similarly, targeting HKT1 transporters for removing Na^+^ from the shoot comes with significant yield penalties because of the high carbon cost for osmotic adjustment (see Shabala *et al*., 2025 for details). The latter strategy is also limited by the relatively small capacity of the root to store excessive Na^+^ without experiencing toxicity symptoms. Targeting tissue tolerance traits appears to be much more promising, given that this mechanism has been evolutionarily proved. Indeed, evolutionary analysis of salt tolerance in angiosperms showed that halophyte species have arisen independently multiple times (Flowers *et al*., 2010), yet their remarkable ability for efficient vacuolar Na^+^ sequestration was preserved on every occasion. How can this trait be targeted in breeding programmes?

The canonical view on vacuolar Na^+^ sequestration is that it is mediated by tonoplast‐based NHX Na^+^/H^+^ exchangers (Apse *et al*., 1999), and overexpression of halophytic PutNHX1 and SeNHX1 in Arabidopsis resulted in a salt‐tolerant phenotype (Liu *et al*., 2017). However, overexpressing the *AtNHX1* gene in barley has not resulted in salt‐tolerant phenotype (Adem *et al*., 2015) as active removal of Na^+^ from the cytosol into the vacuole is only one (of several) component of the vacuolar sequestration. To enable efficient NHX operation, this transporter needs to be energized by tonoplast H^+^ pumps (either H^+^‐ATPase or H^+^‐pyrophosphatase); in addition, Na^+^ back‐leak from the vacuole into the cytosol through tonoplast‐based slow (SV) and fast (FW) vacuolar channels (Shabala *et al*., 2020). Each of these transporters is regulated by numerous factors. For example, H^+^‐ATPase activity is dependent on cytosolic pH and Ca^2+^ concentrations and is regulated by various ligands (with 14‐3‐3 proteins and PKS kinases being prominent examples; Falhof *et al*., 2016). Similarly, H^+^‐PPase activity has strong dependence on K^+^ (Zhou *et al*., 2020), and FV channels are strongly affected by cytosolic Ca^2+^, Mg^2+^, and polyamines (Shabala *et al*., 2020) – all of which are modulated by salt stress. So, how can all these factors be accounted for in the breeding programmes, and where the cell‐based phenotyping steps in?

**Fig. 1:**
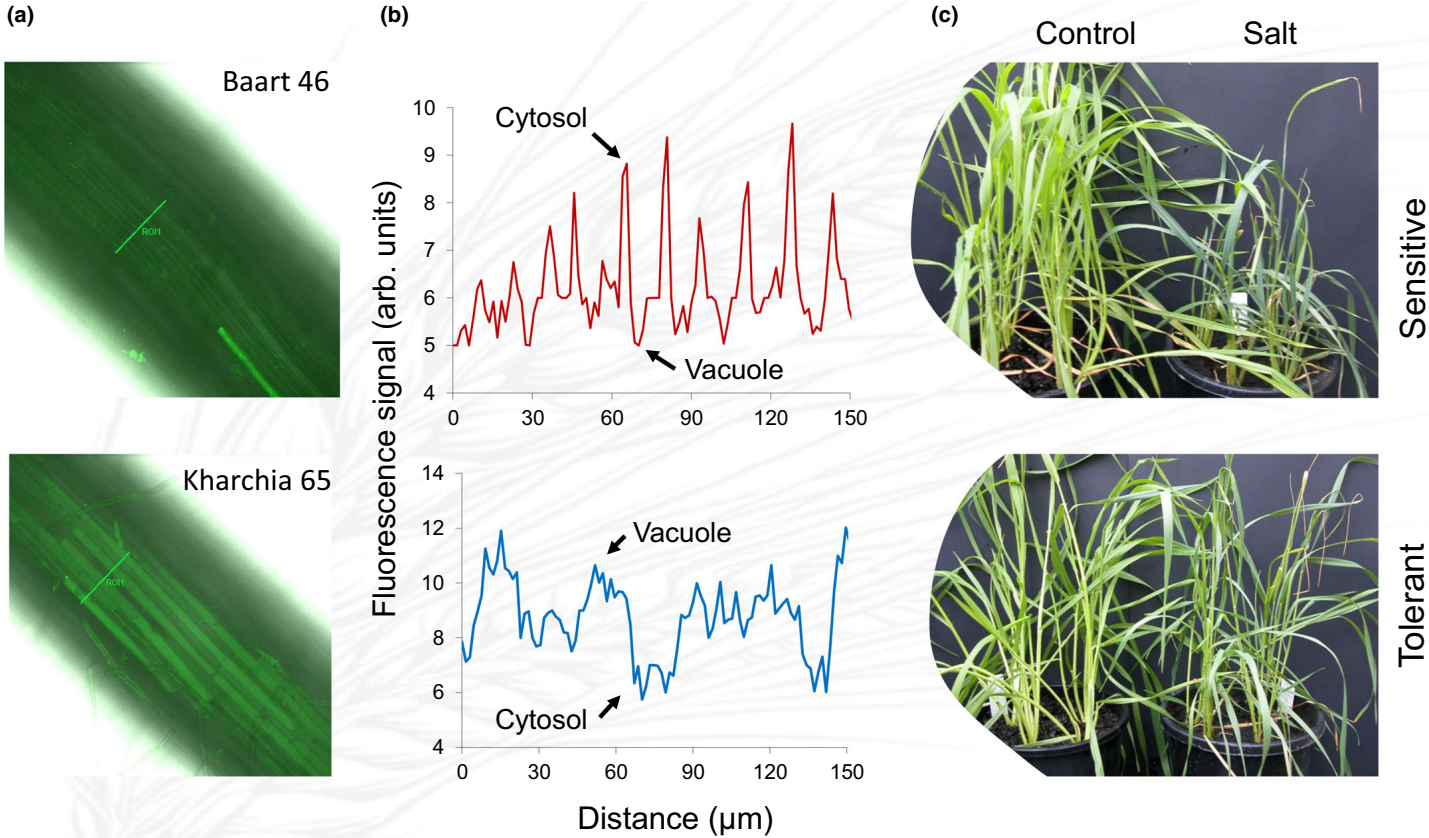
Using a CoroNa green fluorescence probe to quantify the efficiency of vacuolar Na^+^ sequestration in wheat (based on Cuin *et al*., 2011). Two contrasting bread (*Triticum aestivum*) wheat varieties (Baart 46 – sensitive; Kharchia 65 – tolerant) are used as an example. Roots of 6‐d‐old wheat seedling were treated with 150 mM NaCl for 24 h. The 10‐mm‐long segments were then cut from the mature root region. The fluorescence dye was loaded (see Cuin *et al*., 2011 for details), and root segments were washed and examined using confocal microscopy. Images were recorded with a Leica TCS SP5 confocal microscope (Leica Microsystems CMS GmbH, Germany), equipped with an acousto‐optical beam splitter (a) and the intensity of the fluorescent signal was quantified along an arbitrary drawn region of interest (ROI) axis (b). The cytosolic Na^+^ content in the salt‐tolerant Kharchia 65 cultivar is consistently (by *c*. 40%) lower than that of the vacuole. Conversely, in the salt‐sensitive Baart 46 genotype the cytosolic Na^+^ content is *c*. 80% higher than that in the respective vacuole (b). This difference in vacuolar Na^+^ sequestration ability is reflected in differential salt stress tolerance reported in glasshouse experiments after treating plants with salt stress for 4 wk (c).

Several groups have demonstrated that CoroNa Green fluorescence dye may be used as a sensitive tool for characterizing the patterns of intracellular distribution of Na^+^ between various intracellular compartments (Oh *et al*., 2009; Park *et al*., 2009; Cuin *et al*., 2011; Wu *et al*., 2015, 2019). As illustrated in Fig. 1, the intensity of vacuolar and cytosolic CoroNa Green signals is drastically different between contrasting Baart 46 (sensitive) and Kharchia 65 (tolerant) wheat varieties. Thus, one can then create a double‐haploid (DH) population from these contrasting parental lines and then screen them using confocal laser scanning microscopy. Quantitative trait locus (QTL) associated with differential vacuolar Na^+^ sequestration ability may then be identified using an appropriate software package following composite interval mapping (CIM) and multiple‐QTL mapping approaches (Li *et al*., 2008). Molecular markers associated with significant QTL can then be delivered for breeding programmes, and DEGs specifically located within identified QTL regions can be revealed by bioinformatics analysis as candidate genes, following their functional validation. None of this can be achieved by whole‐plant phenotyping that does not allow us to distinguish between vacuolar and cytosolic Na^+^ distribution.

Another example is related to plants' adaptive responses to soil flooding. Soil flooding costs the economy over US$70 billion p.a. in lost production (Voesenek & Sasidharan, 2013), and the frequency and severity of flooding events are only going to increase (Yun & Shabala, 2026). Until now, the efforts of plant breeders were focused on traits related to roots' ability to acquire and/or preserve oxygen, such as formation of aerenchyma, formation of the radial oxygen loss barrier or lateral root development (Daniel & Hartman, 2024; Renziehausen *et al*., 2025). However, oxygen shortage is only one of the constraints affecting plant performance in flooded soils. As elemental toxicity (and, specifically, that of Mn^2+^ and Fe^2+^) arises from the decline in soil redox potential (Marschner, 1995), in addition to their ability to acquire and accumulate O_2_ for metabolic purposes, plants need to possess mechanisms to deal with the accumulation of these elemental toxins to adapt to flooded soils. Recent bioinformatics analysis has revealed that wetland species possessed a larger proportion of predicted gene copies in genomes of several genes and gene families related to heavy metal transport (Yun & Shabala, 2026). Of specific interest were tonoplast‐localized Zinc transporter 1 and plasma membrane‐localized ZIP2 that are related to Zn/Mn transport and sequestration in the root (Milner *et al*., 2013) and tonoplast‐localized metal tolerance protein 8 (MTP8) that is essential for Mn detoxification by subcellular compartmentation (Takemoto *et al*., 2017). How can their functional roles in plant tolerance to soil flooding be assessed, and QTLs conferring tolerance to elemental toxicity be discovered? It is obvious that whole‐plant phenotyping by hyperspectral imaging offers little help here; what about a cell‐based approach?

Earlier, we screened 20 barley varieties for their sensitivity to Mn stress (Huang *et al*., 2015) and showed that waterlogging‐tolerant genotypes TF026 had a 1.7‐fold higher Mn^2+^ concentration in leaves than the sensitive genotype Naso Nijo, suggesting that the former variety was highly efficient in sequestering relatively large amounts of Mn^2+^ in leaves without a significant impact on metabolism. Recently, Kahali *et al*. (2024) reported a novel, water‐soluble, cell‐permeable fluorescent Mn^2+^ selective sensor for visualization of manganese dynamics in live mammalian cells. Thus, one may envisage a scenario for creating and using DH lines made of these contrasting varieties and then utilize them for precise QTL mapping of genes conferring vacuolar Mn^2+^ sequestration by the MTP8 transporter using the above dye.

Similarly, electrophysiological methods and, specifically, the noninvasive microelectrode ion flux estimation (MIFE) technique (Shabala *et al*., 1997, 2006) may be used to reveal QTL conferring key transporters mediating uptake of toxic metals by plants. This may be specifically true for revealing QTL for ZIP2 Mn^2+^ uptake transporter if using recently developed Mn^2+^ liquid ion exchanger (LIX) (Horning *et al*., 2020). Fluxes of other heavy metals such as Cd^2+^ (Hafsi *et al*., 2022) or Pb^2+^ (Li *et al*., 2017) can also be measured. When performed on genetically diverse materials (such as NIL or DH lines), such measurements may allow breeders to discover novel QTL conferring transporters for these genes.

**Fig. 2:**
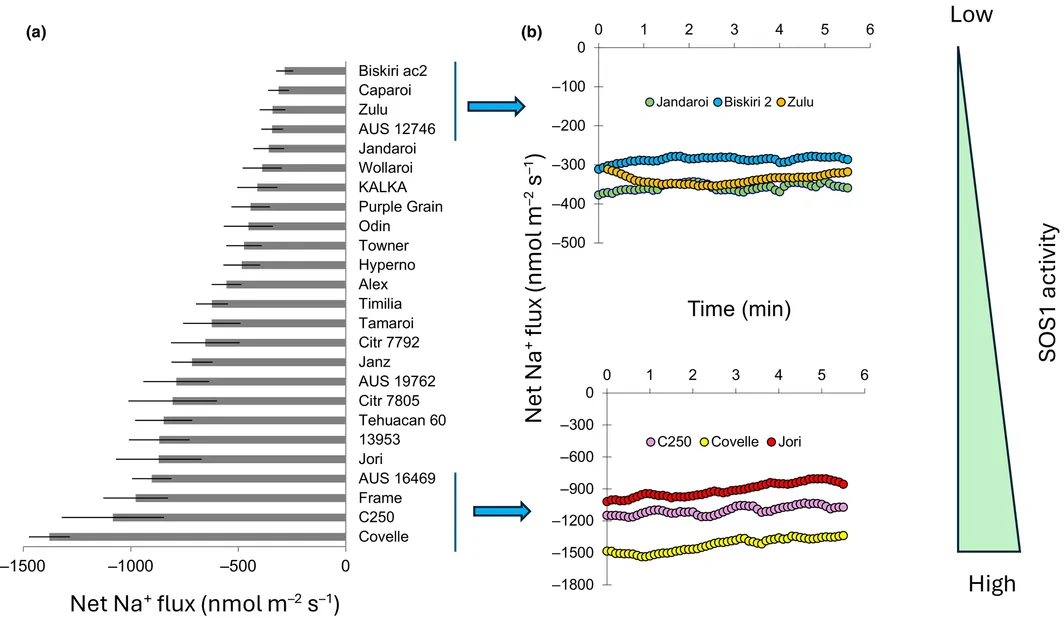
Using noninvasive microelectrode ion flux estimation (MIFE) techniques for the functional assessment of salt overly sensitive 1 (SOS1)‐mediated Na^+^ extrusion ability in durum (*Triticum turgidum* ssp. *durum*) wheat using the so‐called ‘recovery protocol’ (Cuin *et al*., 2011; Wu *et al*., 2019). (a) Illustrates the extent of genetic variability in SOS1‐mediated Na^+^ extrusion among 25 varieties screened (based on Cuin *et al*., 2009). (b) Depicts real‐time recordings on net Na^+^ fluxes from the root apex of six varieties contrasting in their SOS1 operation. Varieties C250, Covelle and Jori had four‐ to fivefold higher SOS1 activity than the less efficient cultivars Jandaroi, Biskiri and Zulu. The entire measurement takes several minutes and is carried out on roots of young (3‐ to 4‐d‐old) seedlings grown hydroponically.

**Fig. 3:**
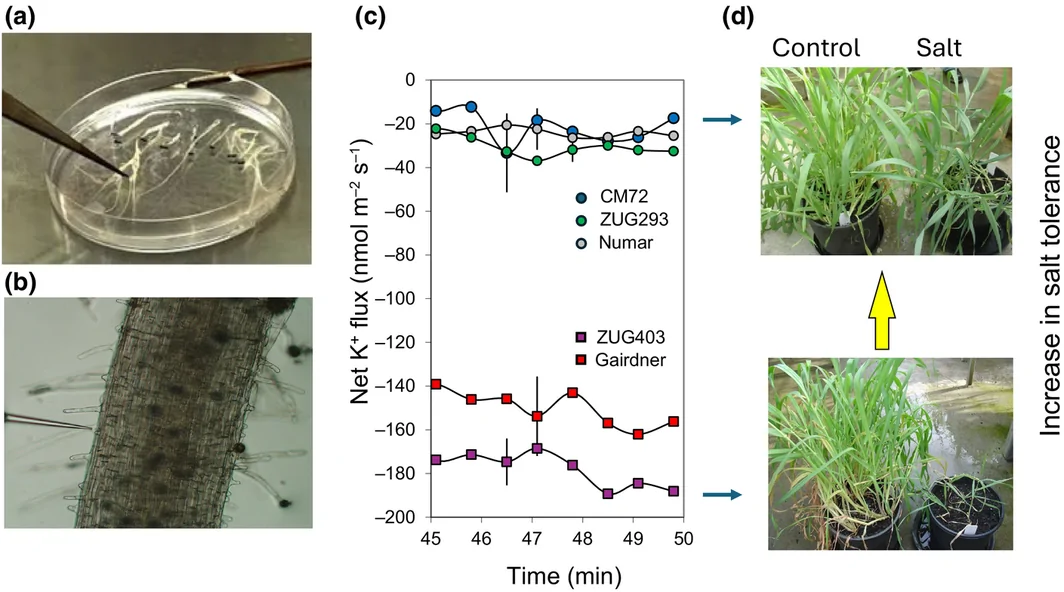
Using noninvasive microelectrode ion flux estimation (MIFE) techniques for the functional assessment of efficiency of root cytosolic K^+^ retention, a key component of salinity tissue tolerance. Mature root segments from 3‐ to 4‐d‐old hydroponically grown barley seedlings are immobilized in Petri dishes (a) and exposed to 80 mM NaCl treatment for 45 min. K^+^‐selective microelectrode is then positioned above the root epidermis (b), and net K^+^ loss is measured for 3–4 min (c). The reported sixfold difference in the magnitude of K^+^ loss between sensitive (ZUG430 and Gairdner) and tolerant (CM72, Numar and ZUG293) reflects differential salt stress tolerance reported in glasshouse experiments after treating plants with salt stress for 4 wk (d). Based on Chen *et al*. (2005).

Electrophysiological MIFE measurements may also be used as a powerful tool for identifying QTL conferring Na^+^ exclusion from uptake. Earlier, we have shown that the amiloride‐sensitive component of net Na^+^ flux measured by calixarene‐based Na^+^ LIX can be used as a reliable proxy for the functional SOS1 activity (Wu *et al*., 2019). When 25 durum wheat varieties were screened for their ability for SOS1‐mediated Na^+^ extrusion, a four to five‐fold difference was reported between varieties (Fig. 2). Thus, this method can be used in the search for efficient SOS1 donors to construct DH populations in various species, followed by their QTL mapping for SOS1 location. The same approach can be used for discovering traits and genes conferring cytosolic K^+^ retention – a key component of salinity tissue tolerance (Wu *et al*., 2018; Rubio *et al*., 2020). In barley, over 60% of genetic variability in salinity tissue tolerance was attributed to the ability of roots to prevent NaCl‐induced K^+^ loss from root epidermis (Chen *et al*., 2005, 2007). The difference in the magnitude of NaCl‐induced K^+^ efflux between contrasting varieties was five‐ to sixfold, with the measurement taking only a few minutes (Fig. 3), making it a highly convenient phenotyping tool. The critical role of root K^+^ retention as a determinant of salinity tolerance in various plant species has been shown by groups (e.g. native grass species – Wang *et al*., 2026; sunflower – Hryvusevich *et al*., 2021; alfalfa – Xiong *et al*., 2018; tomato – Wei *et al*., 2017) validating microelectrode K^+^ efflux measurement as a suitable proxy for cell‐based phenotyping for salinity stress tolerance.

## Current limitations and prospects of cell‐based phenotyping

**Fig. 4:**
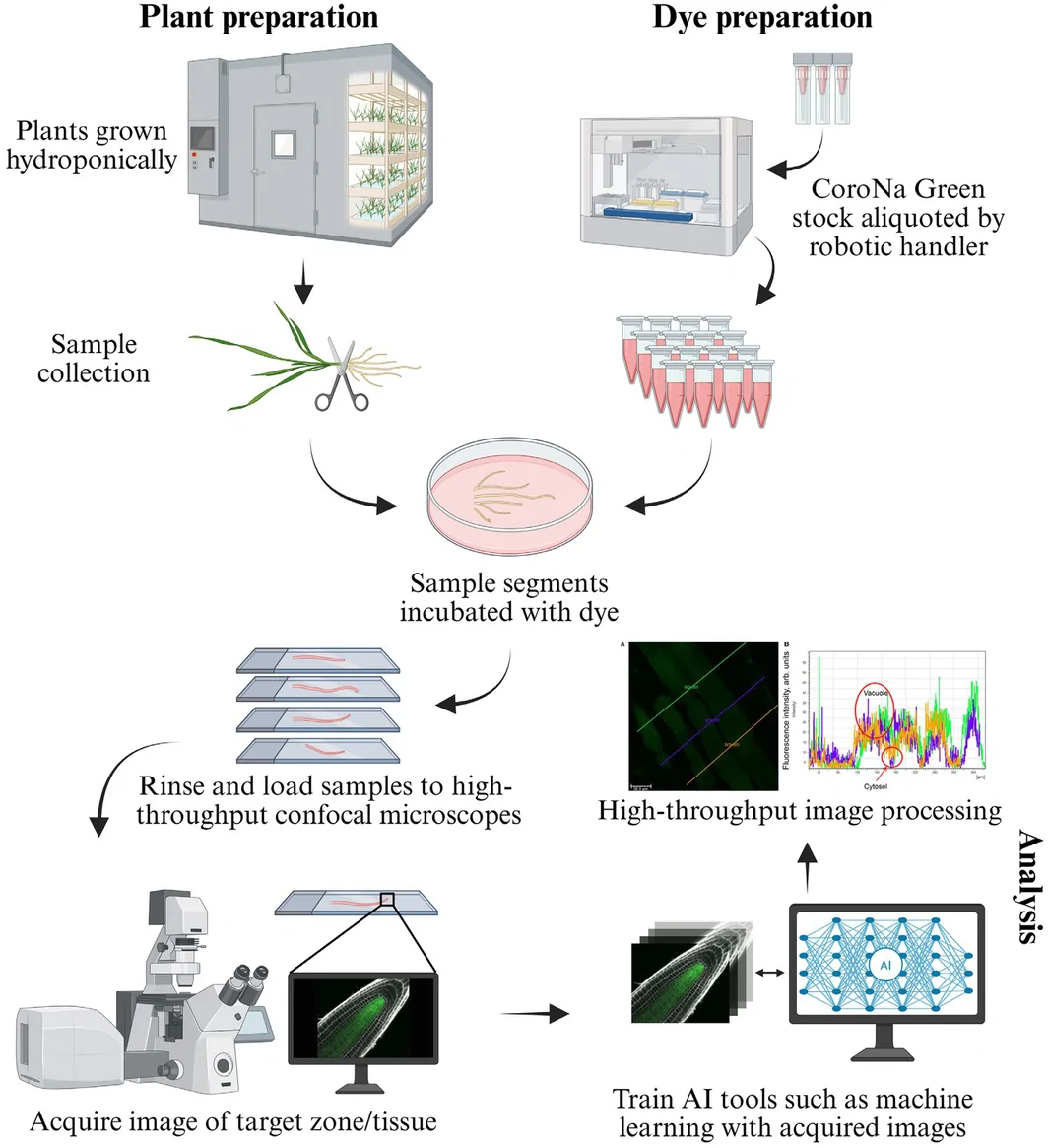
Flow chart illustrating the proposed pathway for improving the throughput of fluorescence imaging‐based phenotyping by using robotics and machine learning (ML) approaches. Fluorescent dyes are aliquoted and prepared, and plants are grown hydroponically and immobilized in measuring chambers. Solutions and dye are loaded, plants are incubated and washed, and specimens are then transferred to the microscopic stage. Most operations are carried out by robotic arms in parallel processing. Using ML and artificial intelligence (AI) tools, required root zones are selected and images acquired and processed/analysed. Created in BioRender by Yun P (2026) (https://BioRender.com/dnjtt9f).

The feasibility of cell‐based phenotyping was demonstrated two decades ago (Chen *et al*., 2005). Nevertheless, despite numerous calls for the paradigm shift towards physiological phenotyping at the cellular/organ level (Ghanem *et al*., 2015; Großkinsky *et al*., 2015; Gill *et al*., 2017), the method still lags the advancements in omics. Several major factors contribute to this low adoption of available technologies. The most critical is the lack of automation and relatively low throughput of proposed methods. Breeders are interested in screening hundreds (ideally, thousands) of genotypes per day, whereas all current electrophysiological and/or imaging methods are unable to enable this. However, the above limitation is merely technical and can be overcome relatively easily by a broader use of automation for specimen handling and image processing (Fig. 4). The analogy can be made with the Nobel Prize‐winning patch‐clamp technique (Ghovanloo *et al*., 2025). Developed in the early 1980s, this method relied on manual specimen handling and establishing gigaohm seals, followed by characterization of ion channel properties. As a result, the throughput capacity of the method was 15–20 specimens per day. As the method gained momentum and became accepted by the pharmaceutical companies as a major tool in drug discovery, automation came in place. Now, the IonWorks Barracuda automated patch‐clamp system runs in parallel 384 channels/amplifiers and allows screening of up to 6000 specimens per day (www.moleculardevices.com/barracuda). There is no technical reason why the same cannot be done with electrophysiological‐ or image‐based methods for single‐cell phenotyping in plants. The major hurdle in this process is a lack of dedicated investment. Right now, medical science receives the ‘lion's share’ of global life sciences funding, dwarfing crop breeding by hundreds of billions of dollars annually. This is hardly surprising as medical research yields direct, immediate human health benefits, while foundational plant sciences – despite being critical for global food security and climate resilience – often struggle to secure equivalent financial and structural support. This calls for a need for robust investment from companies and/or governments. Once this occurs, bringing cellular/tissue‐specific traits into phenotyping will, without a doubt, bridge the gap between molecular and plant levels and will revolutionize crop breeding for abiotic stress tolerance.

As shown above, at this stage, fluorescent probes are the most promising candidates for automation and are applicable to various cell types and species (Ivsic *et al*., 2025). The most serious technical hurdle in the adoption of this approach is a lack of the ratiometric Na^+^ dye that would allow quantification of the absolute Na^+^ concentrations in various intracellular compartments. However, such quantification may not be necessary for selecting promising genotypes, especially given a 15‐fold difference between vacuolar signals from varieties contrasting in salinity stress tolerance (Wu *et al*., 2015). A semi‐quantitative approach is also feasible, using *in vitro* dye calibration in a buffer solution containing different Na^+^ and K^+^ concentrations (Wu *et al*., 2015). In addition, high‐throughput confocal imaging of intact tissue allows detection of cells from the sample surface to a few hundred micrometres underneath (Salomon *et al*., 2010), linking the data with spatial transcriptomics. The protoplast‐based screening, such as microfluidics‐based flow cytometry (Dai *et al*., 2022) and protoplast transactivation system (Wehner *et al*., 2011), has also demonstrated the potential of high‐throughput functional characterization and can be used to match scRNA‐seq data.

Automation of MIFE‐type ion flux measurements is also technically feasible. The cost of electronics is relatively cheap. Thus, using recent advances in microfluidics and the lab‐on‐a‐chip approach (Rothbauer *et al*., 2015; Berlanda *et al*., 2021), one can make an automated system that may enable parallel measurement of ion fluxes for up to 50 specimens. The limiting factor here will be a human factor – for example, the speed with which an operator would be able to immobilize plant specimens. Given that most protocols require only several minutes of flux recording, such a system will be able to assess up to 200 plant samples per hour, or > 1000 specimens per day. While the initial cost may be high, and quick returns also unlikely, the operating costs will be rather small and in the order of cents, as the cost of consumables is almost nonexistent, and the system's operation requires no high qualification.

The merger between cell‐based phenotyping and omics may hardly be possible without developing advanced analytical tools. Therefore, machine learning‐based methods are critical, especially for accurately converting cell images into quantitative phenotype data for high‐throughput analysis pipelines. Good examples include PlantSeg (Wolny *et al*., 2020), MorphoGraphX (Barbier de Reuille *et al*., 2015), Cellpose (Stringer *et al*., 2021), and ilastik (Berg *et al*., 2019). The advent of artificial intelligence (AI) tools may also revolutionize the field. Many proteins are expressed only in specific cell types, and AI‐assisted protein design, especially those with multiple static states, such as secondary active transporters (Drew & Boudker, 2024), requires more physiological data for accurate prediction (Fang *et al*., 2025). Cell‐based phenotyping can bridge this current gap in the knowledge. Overall, it will accelerate AI‐assisted crop design to optimize proteins for better climate change adaptation (Li *et al*., 2025; Zhang *et al*., 2025).

## Competing interests

None declared.

## Disclaimer

The New Phytologist Foundation remains neutral with regard to jurisdictional claims in maps and in any institutional affiliations.

## Supporting information


**Notes S1** Criteria for literature search for omics papers.
Please note: Wiley is not responsible for the content or functionality of any Supporting Information supplied by the authors. Any queries (other than missing material) should be directed to the *New Phytologist* Central Office.
