# Supplementary material for: Cell‐based crop phenotyping for future climates
Source: New Phytol. 2026 Jul 9;252(2):575–87. doi: 10.1111/nph.71397 (PMC13583381; doi:10.1111/nph.71397)
Supplement: Supplementary file 1 [file NPH-252-575-s001.docx]

## ***New Phytologist* Supporting Information**

Article title: Cell-based crop phenotyping for future climates

Authors: Sergey Shabala, Ping Yun, Zhong-Hua Chen, Meixue Zhou, Min Yu, and Nadia Bazihizina

Article acceptance date: 11 June 2026

**Supporting information – Criteria for Literature Search for Omic Papers**

The search was performed on the 9 June 2026 with the query string (TITLE-ABS-KEY ( human OR humans OR mammal* OR mammalian ) AND TITLE-ABS-KEY ( "single-cell omics" OR "single cell omics" OR "single-cell multiomics" OR "single cell multiomics" OR "single-cell transcriptomics" OR "single cell transcriptomics" OR "single-cell genomics" OR "single cell genomics" OR "single-cell epigenomics" OR "single cell epigenomics" OR "single-cell proteomics" OR "single cell proteomics" OR scRNA-seq OR "single-cell RNA sequencing" OR "single cell RNA sequencing" OR "single-cell sequencing" OR "spatial omics" OR "spatial transcriptomics" OR "spatial proteomics" OR "spatial metabolomics" OR "spatial multiomics" )). The results were subsequently filtered to include research articles only. For the plant-focused search, the organism-related terms were replaced with TITLE-ABS-KEY ( plant* OR arabidopsis ), and the single-cell and spatial omics queries were run separately.
